# Use of Taurolidine in a Patient With a Cardiac Implantable Electronic Device Protrusion

**DOI:** 10.1016/j.jaccas.2023.101835

**Published:** 2023-04-19

**Authors:** Marcello Giudice, Bruna Catuzzo, Nicola Berlier, Ernest W. Lau, Hendrik Bonnemeier, Ojan Assadian, Benito Baldauf, Stefan Borov, Paolo Scacciatella

**Affiliations:** a Department for Cardiology and Electrophysiology, U. Parini Hospital, Aosta, Italy; b Royal Victoria Hospital, Belfast, United Kingdom; c Helios Klinikum Cuxhaven, Cuxhaven, Germany; d Helios Klinikum Wesermarsch, Nordenham, Germany; e Christian-Albrechts University, Kiel, Germany; f Lakumed Kliniken, Landshut, Germany; g Landesklinikum Wiener Neustadt, Wiener Neustadt, Austria

**Keywords:** cardiac implantable electronic device, fistula, infection, lead extraction, salvage, skin erosion, taurolidine

## Abstract

We report the successful salvage of cardiac implantable electronic device pulse generator protrusion sealed by the surrounding skin in a frail patient presenting 5 months after the last surgical revision. (**Level of Difficulty: Advanced.**)

## Graphical abstract

**Figure:**
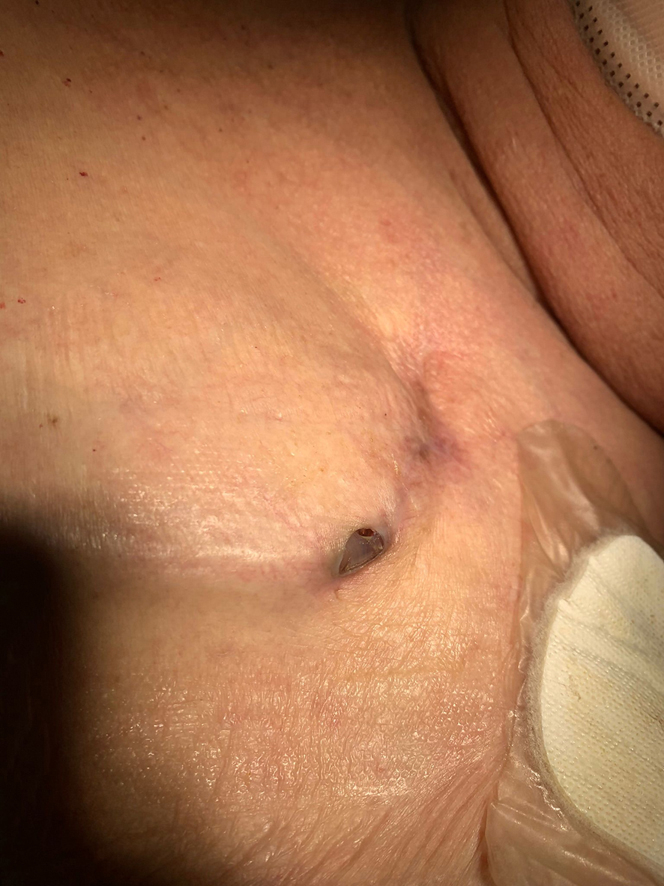

## History of Presentation

**Figure 1:**
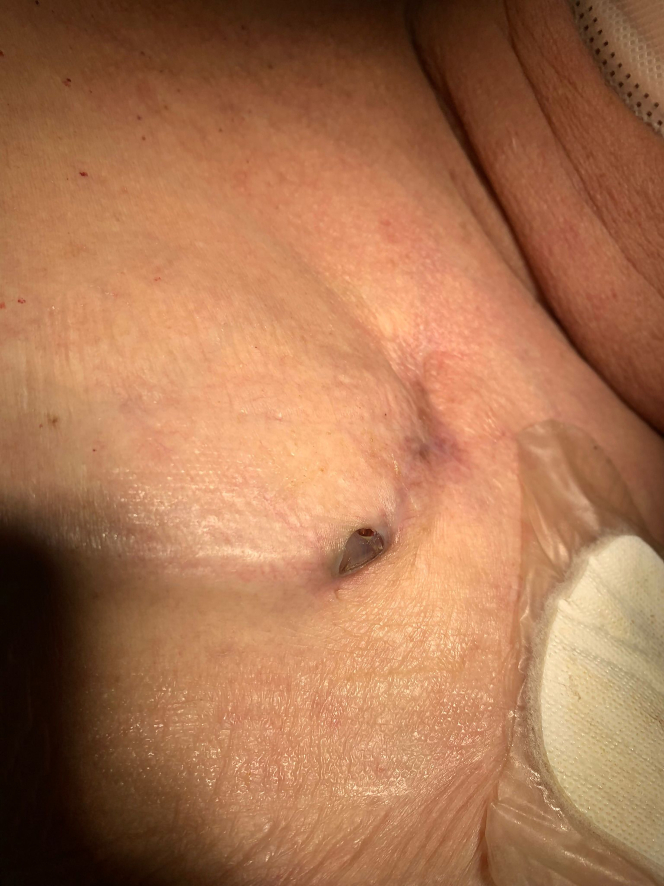
Clinical Aspect Pulse generator protrusion through skin erosion 5 months after upgrade revision.

A 77-year-old man with dilated cardiomyopathy and permanent atrial fibrillation was upgraded from a single-chamber implantable cardioverter defibrillator to a cardiac resynchronization therapy defibrillator system with insertion of a new left ventricular lead due to deterioration of the left ventricular ejection fraction (LVEF), which was 28% at the time of the upgrade and had recovered to 50% during the most recent ultrasound study. There were no acute intraoperative or postoperative complications. Five months later, he presented with partial protrusion of the cardiac implantable electronic device (CIED) pulse generator through a small skin defect at the lateral corner of the original incision (Figure 1). The skin defect formed a “tight” seal around the exposed corner of the CIED pulse generator. The surrounding skin looked healthy and not inflamed or infected. The patient was clinically well with no symptoms.

The patient was started on empirical Amoxicillin/Clavulanic acid therapy and investigated as per local policies and international guidelines.^1^

**Learning Objectives**

• To distinguish between different types of CIED infection and their recommended therapy.
• To learn about treatment options for CIED infection in frail patients and those who are unwilling to consent to a conservative approach or guideline-recommended therapy.

## Medical History

The patient underwent his first implantation at age 68 years with an implantable cardioverter defibrillator due to syncopal events and dilated cardiomyopathy with slightly impaired LVEF of 48% during cardiac ventriculography and in absence of coronary vessel disease. The patient was experiencing chronic renal insufficiency IIIb and permanent atrial fibrillation, which had been detected due to ischemia in the splanchnic area with subsequent partial resection of the colon. At that time the patient was on optimal medical treatment for heart failure and renal impairment.

## Investigations

Swabs around the skin defect and 3 sets of blood cultures drawn from different sites did not grow any microbes. Blood tests did not suggest systemic infection. Transthoracic and transesophageal echocardiography showed no vegetations. There was no liquid retention in the area of the cardiac resynchronization therapy defibrillator system pocket, or purulent discharge from the skin lesion.

## Management

Complete system explantation with lead extraction was proposed to the patient as per the current guidelines but rejected.^1^^,^^2^ After evaluation of the alternative management strategies, the patient and his medical team agreed on an attempt at system salvage in preference of negative pressure therapy^3^^,^^4^ and/or chronic antibiotic suppression.^5^

**Figure 2:**
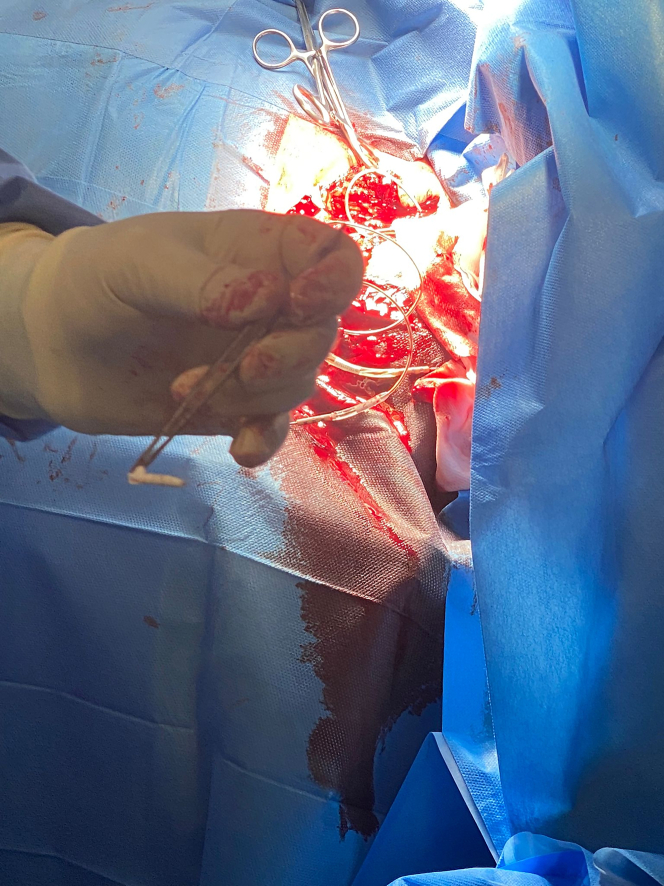
Revision Suture fixation sleeves and all nondegradable materials identified were removed.

**Figure 3:**
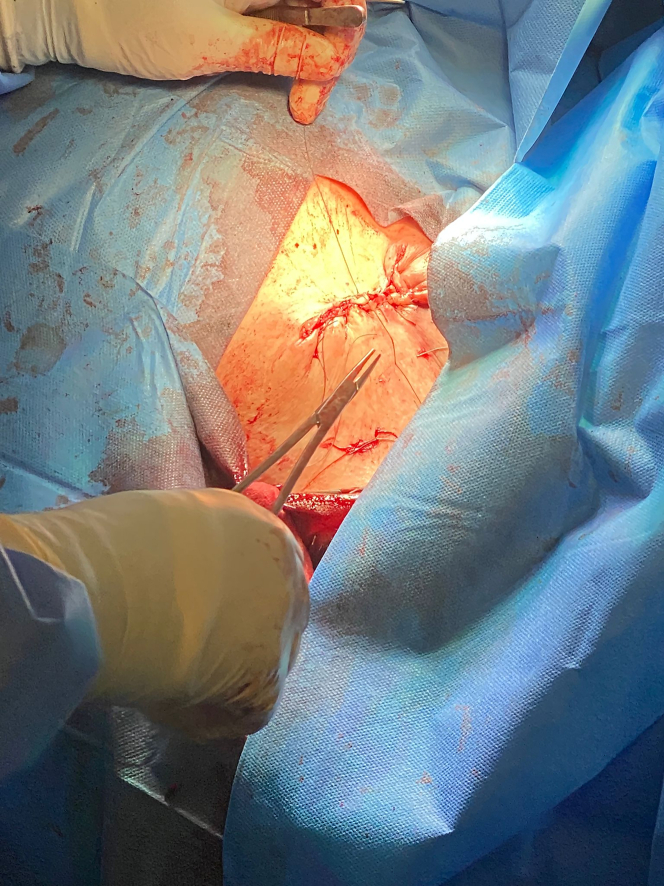
Closure Wound closure after revision procedure.

During the salvage procedure, an island of skin incorporating the incision scar and the erosion defect was excised. The CIED pulse generator was extracted out of its capsule and disconnected from the leads. The pocket (with the old leads in place) was then irrigated with TauroPace (TP) (TauroPharm), a taurolidine based antimicrobial solution specifically for use in cardiac device procedures.^6^ The CIED pulse generator was wrapped in a swab and submerged in 100 mL of TP for the entire 120 min of the revision procedure. All nonabsorbable sutures, anchor sleeves, and foreign materials except the implanted leads were removed (Figure 2). The entire fibrous capsule, including the calcified fibrous tissues encasing the extravascular segments of the leads, was then excised (ie, capsulectomy). The surgical site was irrigated with another 50 mL of TP, with the extravascular segments of the leads immersed in the solution. A new subpectoral pocket was fashioned under the former prepectoral pocket and thoroughly irrigated with TP. The connector pins were wiped with swabs soaked with TP before being plugged back into their respective ports on the old pulse generator. The torque wrench was dipped in TP before piercing the grommets covering the set screws. No new anchor sleeves were applied. All the hardware was positioned within the newly fashioned subpectoral pocket, which was instilled with another 20 mL of TP before closure. The prepectoral pocket was then closed up with interrupted sutures (Figure 3). The wound was dressed and pressure dressing was applied over the pocket.

## Discussion

CIED components (pulse generator, anchor sleeves, leads) may protrude out of the device pocket if the lining tissues (typically skin and subcutaneous tissues) break down. The pocket breach typically occurs through the tissues lining the edges rather than covering the surfaces of the pulse generator for several reasons. First, the edge lining tissues are more stretched than the surface lining tissues, becoming thinner and more at risk of pressure-induced necrosis. Second, the capillaries running through the edge lining tissues may be compressed, reducing their blood supply (ie, pressure-induced ischemia).^7^ Third, the edge lining tissues are more exposed to mechanical abrasion by any rubbing over the CIED pocket. This might be one reason current guidelines recommend (weak) implantation of the CIED in the submuscular position because complications seem to occur less frequently.^8^ In our case and when pressure-induced necrosis of the tissue over the generator is suspected, we considered reimplantation of the generator in submuscular position as mandatory.

Infection may contribute to the breakdown process by weakening the lining tissues from within and without.^9^ Once the lining tissues have been breached, the protruded CIED components will be colonized by bacteria. However, the lining tissues around the breach may form a “seal” around the protruded CIED components and stop bacterial colonization from extending into the interior of the device pocket and the unprotruded CIED components.

This possibility opens up the tantalizing prospect of successfully salvaging (in the sense of total internalization with no subsequent infection) a CIED system that has protruded out of the device pocket by pocket revision and CIED component sterilization without complete explantation (which would include lead extraction^8^). In cases with localized CIED infection, it may be possible to achieve a resolution of clinical infection by applying the known principles of the bacterial (pathogenical) foreign body interface with meticulous surgical technique in removing any potentially infectious tissues, including the entire capsule, the anchor sleeves, and all nonabsorbable suture materials that might provide nidi for pathogens to multiply.

Whereas in device-associated endocarditis, antimicrobial therapy follows clear guideline recommendations ^8^ including adjustment of the therapy after pathogen detection from swabs, tissue samples, or blood cultures, initial empiric antibiotic therapy in case of generator protrusion with suspected, localized pocket infection is recommended to cover the usual spectrum of pathogens, which most commonly are coagulase-negative staphylococci among others.^8^ The empirical antibiotic therapy should take into account the local resistance situation, especially with regard to methicillin-resistant Staphylococcus aureus prevalence.^8^

As per local policies and international guidelines, we decided on the antibiotic therapy with Amoxicillin/Clavulanic acid 3 g/day, for a total of 7 days. It has excellent bioavailability, both parenterally and orally.

Device salvage has been attempted in the past, but the clinical experience has hitherto been disappointing (>50% infection recurrence rate).^10^ On the other hand, lead extraction requires special equipment and expertise, may not be readily available, and is associated with significant morbidities and even mortality.^11^, ^12^, ^13^ These factors might tip the benefit:risk balance in favor of CIED system salvage over explantation in specific patients.

In our case, the improvement in LVEF showed a good response to therapy in a short period of time (5 months). The combination of pacemaker dependency, long dwell time of the shock coil with extensive adhesions in the superior vena cava, and comorbidities (renal impairment, permanent atrial fibrillation, ischemia in the splanchnic area) suggested a high risk with guideline-compliant therapy.

Revision of CIED infection with the use of different antimicrobial solutions has been reported before.^14^ Their role in the reported success remains unclear. A specifically developed taurolidine based antimicrobial solution enables in vivo and ex vivo sterilization of biological tissues and CIED hardware throughout the revision procedure, and promotes wound healing through its metabolite taurine during recovery.^15^ These aspects could make the difference.

## Follow-up

**Figure 4:**
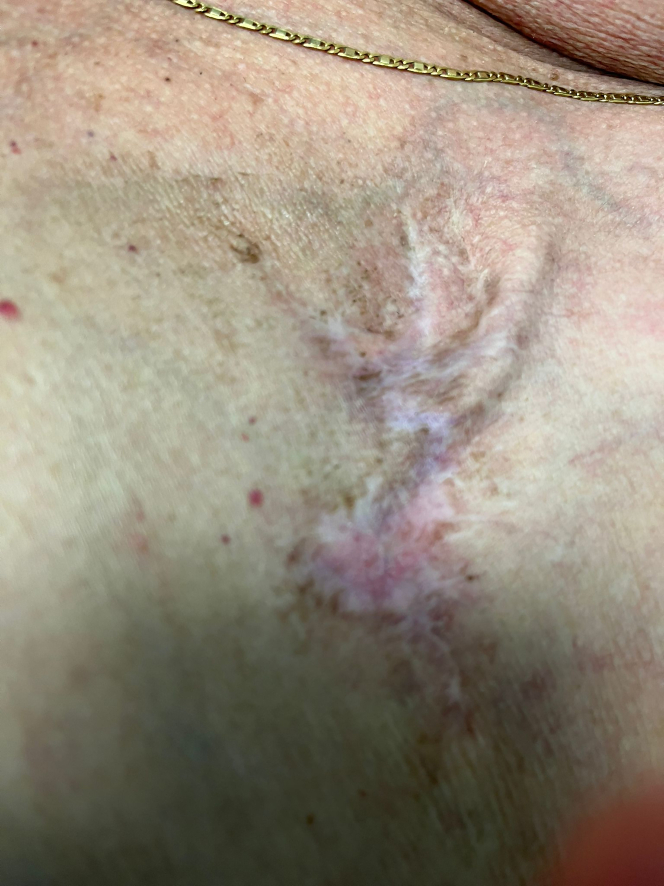
Follow-Up Skin aspect during 12-month follow-up visit at the authors’ center.

Recovery from the procedure was uneventful. During follow-up, there were no clinical or laboratory signs of relapse of infection at 1, 6, 9, and 12 months (Figure 4).

## Conclusions

Although an externally exposed CIED system can be successfully salvaged, the circumstances leading to the adoption of this unconventional management strategy in this case are unusual and system salvage should not be regarded as a general substitute for the standard of care for CIED infection, which is complete hardware explantation. The risks and benefits of any treatment, particularly when deviating from guidelines, should be weighed carefully in the context of each individual patient. However, if the therapeutic objective is medium-term clinical freedom from infection without hardware removal, the salvage procedure described would be a valuable addition to the armamentarium of measures for dealing with CIED infection.

## Funding Support and Author Disclosures

Dr Baldauf is a medical consultant/advisor for the manufacturer of TauroPace. Dr Borov received a research grant through his employer in relation to another manuscript studying the impact of TauroPace in rhythm surgery. All other authors have reported that they have no relationships relevant to the contents of this paper to disclose. Ethical review and approval were waived for this case. According to the current Italian version of the Italian Data Protection Authority article 110 paragraph 1 and European Union General Data Protection Regulation recital 26, the principles of data protection do not apply. Written informed consent has been obtained from the patient to publish this paper.
